# Resveratrol as a Bioactive Compound for Edible Fish Muscle Quality: Emerging Evidence, Putative Mechanisms, and Future Perspectives

**DOI:** 10.3390/foods15162804

**Published:** 2026-08-11

**Authors:** Xiao-Zheng Yu, Yang Yu, Jian-Wei Lin, Jian-Chang Jin, Zi-Yan Liu

**Affiliations:** 1 Zhejiang International Joint Laboratory on Low-Carbon Pollution Control and Resource Utilization, College of Biological and Environmental Engineering, Zhejiang Shuren University, Hangzhou 310015, China; 601824@zjsru.edu.cn (X.-Z.Y.); linjianwei@zjsru.edu.cn (J.-W.L.); 2 State Key Laboratory of Biocontrol, Guangdong Province Key Laboratory for Aquatic Economic Animals, Guangdong Provincial Engineering Technology Research Center for Healthy Breeding of Important Economic Fish, School of Life Sciences, Sun Yat-Sen University, Guangzhou 510275, China; yuyang65@mail2.sysu.edu.cn

**Keywords:** polyphenol metabolism, fillet physicochemistry, flavor precursors, oxidative stability, sensory validation

## Abstract

Resveratrol is a plant-derived stilbene polyphenol that has attracted interest as a potential dietary additive for improving edible fish muscle quality. This review summarizes current evidence on pre-harvest dietary resveratrol supplementation and its possible effects on nutritional composition, flavor-related biochemical traits, and physicochemical quality of fish muscle or fillets. We distinguish nutritional quality, including proximate composition, amino-acid profiles, and fatty-acid composition, from flavor-related and sensory-relevant proxies, such as free amino acids, inosine monophosphate, volatile precursors, color, pH, water-holding capacity, and texture. Direct evidence from edible fish muscle remains limited and fragmented, and most available studies rely on biochemical or instrumental endpoints rather than trained-panel sensory evaluation or consumer acceptance testing. Reported effects suggest species- and dose-dependent changes in muscle protein deposition, amino-acid accumulation, fatty-acid composition, pH, cooking or drip loss, and texture-related traits. Putative mechanisms may involve antioxidant protection, muscle fiber remodeling, lipid metabolism, endoplasmic-reticulum stress, and delivery-dependent bioavailability; however, many mechanistic links are still inferred from hepatic tissues, mammalian models, or in vitro systems. Future studies should combine standardized dose–response feeding trials with gas chromatography–mass spectrometry (GC–MS)-based volatile profiling, lipid and protein oxidation indices, instrumental texture analysis, sensory evaluation, and shelf-life testing before resveratrol can be recommended as a robust feed-based strategy for improving edible fish muscle quality.

## 1. Introduction

With the global population soaring past 8 billion, the surging demand for high-quality animal protein has propelled aquaculture to the forefront of global food security efforts. In the past five decades, aquaculture has experienced a remarkable expansion. According to the latest Food and Agriculture Organization (FAO) report (The State of World Fisheries and Aquaculture 2024), global aquaculture production reached 120 million tons in 2022. This production accounts for 52% of the total seafood supply, representing a nearly 400% increase since 1990. This industry not only furnishes around 20% of the daily protein intake for approximately 3 billion individuals but also eases the pressure on wild fishery resources and generates substantial employment opportunities. As a primary source of high-quality animal protein, fish muscle provides essential nutrients for human diets. However, it is imperative to recognize the fundamental structural and biochemical differences between fish muscle and terrestrial livestock or poultry meat. Structurally, fish muscle is organized into myotomes separated by thin connective tissue sheets (myocommata), lacking the extensive and highly cross-linked collagen network (epimysium, perimysium, and endomysium) characteristic of mammalian and avian muscle. Biochemically, while both provide a complete profile of essential amino acids, fish muscle proteins are inherently more susceptible to rapid postmortem degradation and denaturation. Furthermore, the lipid profile of fish muscle is uniquely dominant in highly oxidation-prone long-chain polyunsaturated fatty acids (LC-PUFAs). Consequently, its nutritional value, flavor, and physicochemical properties are far more frequently and severely compromised by oxidative stress than terrestrial meats, underscoring the urgent need for bioactive compounds to preserve and enhance aquatic muscle food quality.

Intensive aquaculture conditions, including high stocking density, nutritionally imbalanced diets, and environmental stressors, can disturb redox homeostasis in farmed fish [1,2]. Oxidative stress develops when reactive oxygen species (ROS) generation exceeds endogenous antioxidant defenses [3]. In fish muscle, this imbalance can promote lipid and protein oxidation, alter myofibrillar protein structure, and impair water-holding and textural properties [4]. Fish muscle mitochondria can generate ROS under acute stress and may contribute to downstream oxidative damage, although the magnitude of these effects depends on species and stress conditions [5]. Consequently, dietary strategies that strengthen redox homeostasis may help protect edible muscle quality.

Resveratrol (3,5,4′-trihydroxy-trans-stilbene) is a naturally occurring polyphenol abundant in grape skin, peanuts and several berries. It is generally recognized as safe, non-toxic at nutritionally relevant doses, and does not impair zootechnical performance in fish [6,7]. Beyond its classical role as a ROS scavenger, resveratrol modulates sirtuin-1 (SIRT1) [8,9], adenosine monophosphate-activated protein kinase (AMPK) [9], and nuclear factor erythroid 2–related factor 2 (Nrf2) signalling [10,11], thereby orchestrating antioxidant, anti-inflammatory and metabolic reprogramming responses. Consequently, resveratrol has been widely explored in aquafeeds to enhance growth, immunity and resistance to environmental stressors [7,12,13,14,15].

Although recent reviews have collated evidence on the broad physiological impacts of resveratrol in fish, including hepatic metabolism, reproductive performance, and disease resistance [7,16], a critical synthesis focused on edible fish muscle quality remains limited. The present review therefore evaluates current evidence on the effects of pre-harvest dietary resveratrol supplementation on nutritional, flavor-related, and physicochemical attributes of edible fish muscle assessed at harvest or shortly postmortem. Because available data are still sparse, species-specific, and often derived from indirect evidence, this article is framed as an emerging-evidence and mechanistic-perspective review rather than as a conclusive assessment of established fillet-quality benefits. Post-harvest processing technologies, long-term storage systems, and commercial shelf-life interventions are discussed only when they help interpret fish muscle quality indicators; they are not the primary focus of this review.

## 2. Resveratrol

### 2.1. Source and Physicochemical Properties of Resveratrol

**Figure 1:**
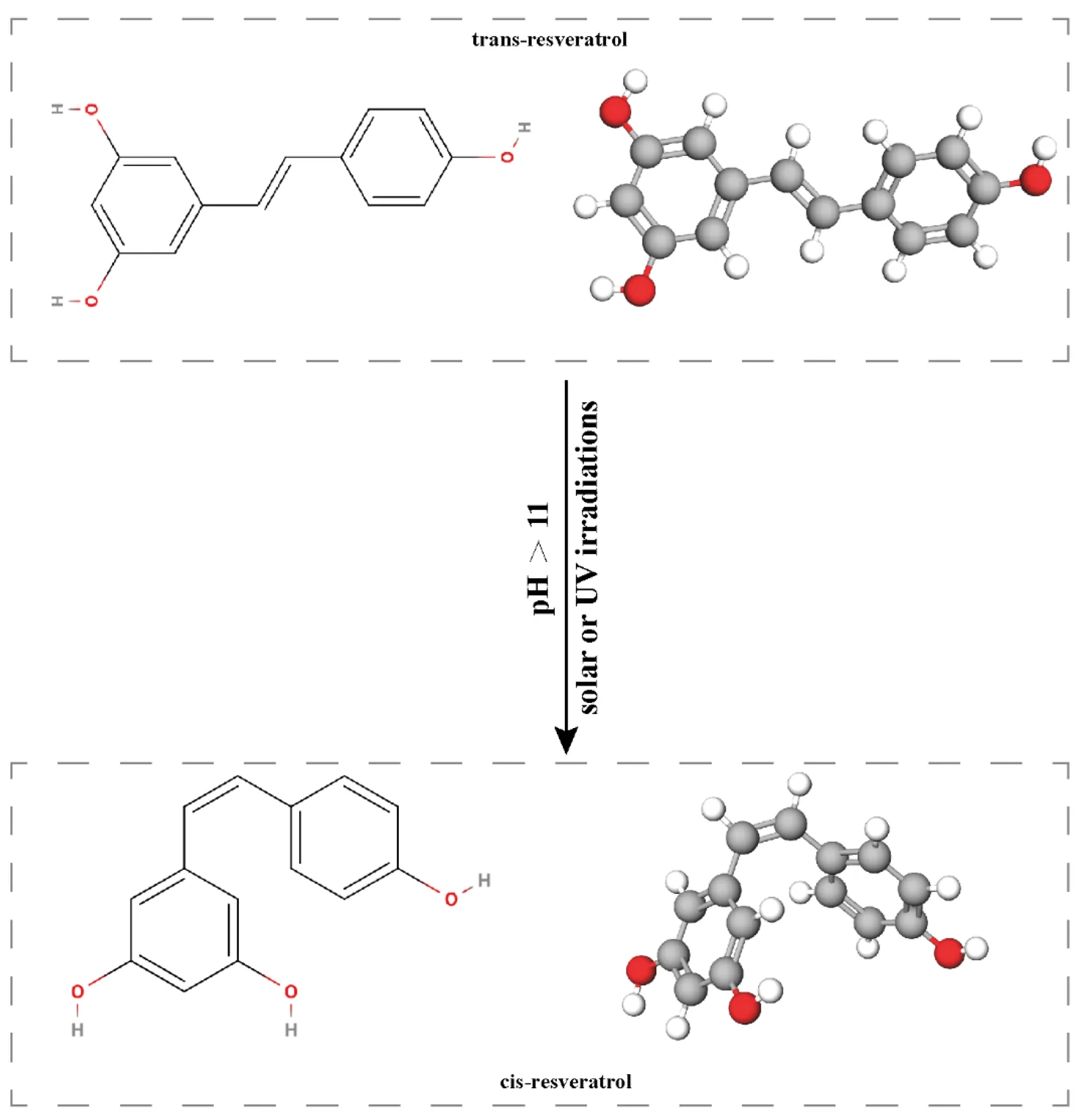
Structures of trans-resveratrol (**top**) and cis-resveratrol (**bottom**).

Resveratrol is a non-flavonoid stilbene polyphenol occurring mainly in grapes, peanuts, berries, and *Polygonum cuspidatum*. It exists mainly as *trans-* and *cis*-isomers (Figure 1), with trans-resveratrol generally regarded as the more stable and biologically relevant form. In plant sources, resveratrol may also occur as the glycosylated derivative polydatin [17,18], which differs from the aglycone in solubility, absorption, and metabolic behavior. Because resveratrol is poorly soluble in water and sensitive to light and alkaline conditions, extraction, storage, feed processing, and delivery systems may influence its stability and biological availability [19,20].

### 2.2. Synthesis and Metabolism of Resveratrol

**Figure 2:**
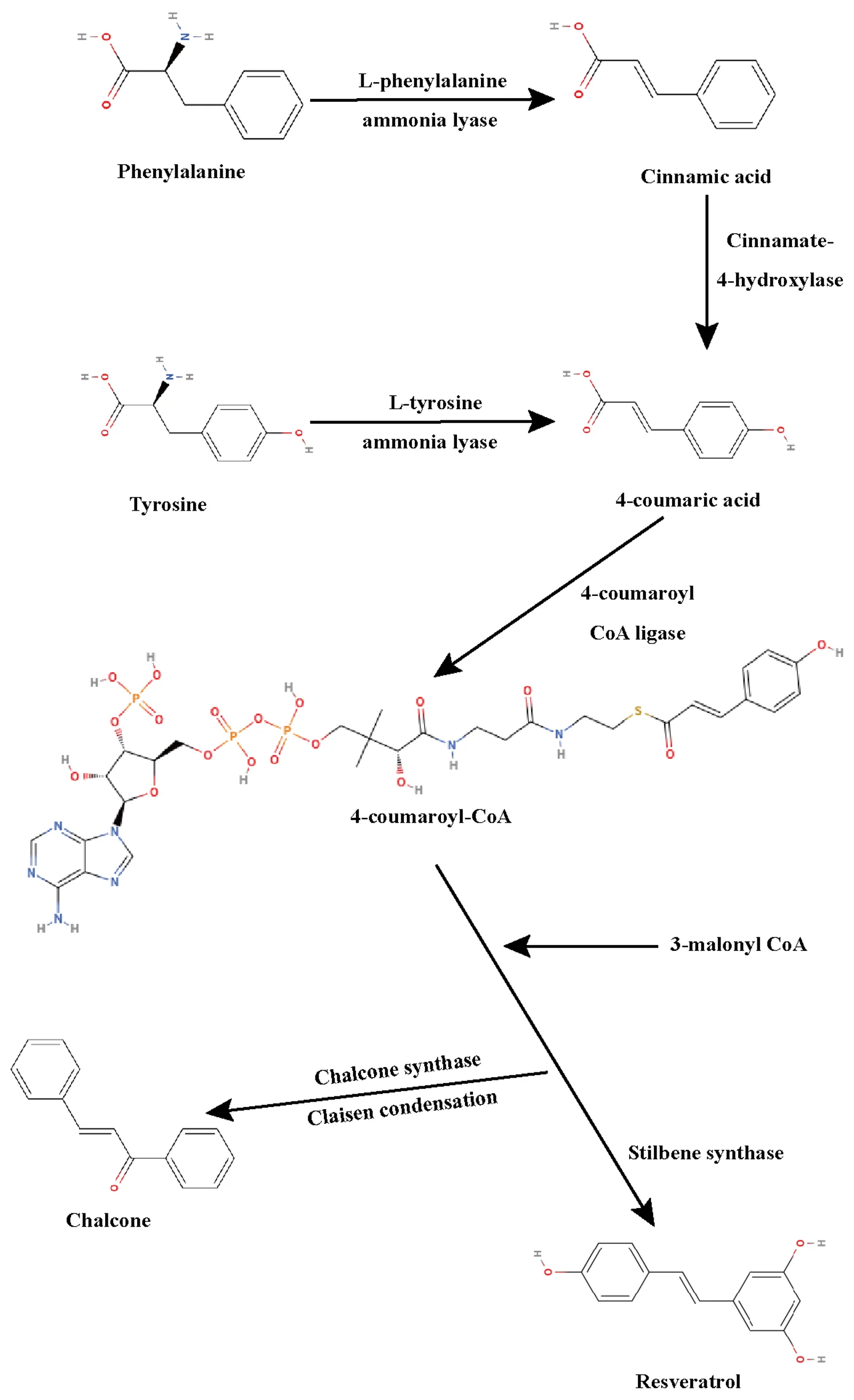
Resveratrol synthesis by the phenylpropanoid pathway.

As a stress-resistant non-flavonoid polyphenolic compound, resveratrol is generally synthesized via the phenylpropanoid metabolic pathway under adverse environmental conditions [21,22]. In plants, de novo biosynthesis of resveratrol primarily occurs via two pathways [23,24,25]: one using phenylalanine as the substrate and the other using tyrosine as the substrate (Figure 2). The biosynthetic process can be summarized as follows: (1) The starting material, phenylalanine or tyrosine, is converted into the central metabolic intermediate *p*-coumaric acid under the catalysis of phenylalanine ammonia-lyase (PAL) and cinnamate-4-hydroxylase (C4H), or tyrosine ammonia-lyase (TAL). (2) *p*-Coumaric acid is then converted to *p*-coumaroyl-coenzyme A (CoA) by the action of the 4-coumarate:CoA ligase (4CL). Subsequent catalysis by different downstream enzymes directs the intermediate into various structural metabolic pathways. (3) Finally, under the catalysis of resveratrol synthase, malonyl-CoA and *p*-coumaroyl-CoA undergo a series of condensation reactions to ultimately form resveratrol [25,26,27].

Currently, resveratrol can be obtained through plant extraction, chemical synthesis, and biotechnological production. Plant extraction, a classical method for resveratrol production, is widely applied in current research; however, the content of resveratrol in plant sources is generally low and is strongly influenced by growth conditions, season, and geographical origin [28,29]. Chemical synthesis involves complex pathways, yields relatively low total product, and generates numerous by-products [30], which limit its industrial application. In contrast, biotechnological approaches offer significant potential for low-cost production and the utilization of renewable resources. In recent years, microbial systems, such as *Escherichia coli*, have been developed for resveratrol biosynthesis [26]. For instance, it has been reported that endophytic fungi isolated from various types of grapes and *Polygonum cuspidatum*, the primary plant sources of resveratrol, possess the ability to produce resveratrol [31]. Exploiting such endophytic fungi may maximize yield and ensure a stable supply.

Animal and clinical studies have yielded inconsistent and controversial findings regarding the efficacy of resveratrol. This is primarily attributed to its rapid metabolism and elimination in vivo, which results in extremely low systemic bioavailability [32]. In humans, oral administration often results in only trace amounts of un-metabolized resveratrol being detectable in the plasma [33]. It is quickly absorbed, metabolized, and excreted, making it difficult to maintain therapeutic plasma concentrations and resulting in poor metabolic stability [34]. This may be attributable to extensive phase II glucuronidation and sulfation metabolism [33,35]. Under normal conditions, resveratrol is absorbed via the gastrointestinal tract and metabolized into glucuronides, sulfates, and hydroxylated derivatives. Human studies show that within 2 h of ingestion, resveratrol is metabolized into 3,4′-O-sulfate and 3-O-glucuronide conjugates [36]. The liver is the primary organ for resveratrol metabolism, and its metabolites are mainly excreted in urine, resulting in a short metabolic cycle [37,38]. Following gastrointestinal absorption, resveratrol has a half-life of only 8–14 min [39], indicating rapid elimination and minimal accumulation in the body.

In the bloodstream, free resveratrol can bind to plasma proteins such as albumin and lipoproteins. It may also undergo enterohepatic recirculation or be rapidly excreted via the urinary system. Ultimately, although resveratrol can be efficiently absorbed after oral intake, the systemic oral bioavailability of unchanged resveratrol is very low, often estimated at less than 1%, mainly because of rapid intestinal and hepatic phase-II metabolism, including glucuronidation and sulfation [40,41]. To overcome these physiological limitations and achieve effective tissue concentrations, innovative formulation and delivery strategies are required, which are discussed in detail in Section 5.

## 3. Effects of Resveratrol on Nutritional, Flavor-Related, and Physicochemical Quality of Fish Muscle and Fishery Products

For clarity, this review distinguishes nutritional quality from sensory quality. Nutritional quality refers to edible muscle composition, including protein, lipid, amino-acid profiles, and health-promoting fatty acids. Sensory quality refers to consumer-perceived attributes such as appearance, aroma, taste, texture, and overall acceptability. Because direct sensory-panel evidence for resveratrol-fed teleosts remains scarce, this section uses the more precise terms flavor-related quality and physicochemical quality when discussing biochemical or instrumental proxies, including fatty acid composition, free amino acids, inosinic acid (IMP), meat color, pH, water-binding capacity, shear stress, and texture [4,42].

Despite these specific variations, general methodological trends can be observed across the literature. In almost all the in vivo teleost studies reviewed herein, resveratrol is uniformly administered as a dietary supplement incorporated directly into formulated aquafeeds. Regarding application parameters, researchers predominantly utilize commercially available, high-purity trans-resveratrol powder, which is homogeneously mixed into the basal diet constituents prior to pelleting or lipid-coating. In terms of developmental stages, the majority of nutritional interventions target the juvenile or active grow-out phases of fish. These stages represent critical physiological windows for robust somatic growth, active muscle fiber recruitment (both hyperplasia and hypertrophy), and intramuscular lipid deposition, making them highly responsive to nutritional programming. Furthermore, to achieve the desired biochemical and physicochemical improvements in muscle tissue, feeding durations in the current literature typically range from 4 to 12 weeks. This timeframe has proven generally sufficient for dietary resveratrol to effectively modulate target metabolic pathways, such as endogenous antioxidant defenses and lipid metabolism, and subsequently translate into measurable enhancements in final fillet quality [43,44,45].

To address the heterogeneity of the cited literature and to make the evidence base more transparent, a structured summary is provided in Table 1. This table distinguishes direct teleost muscle evidence from indirect or extrapolated evidence and classifies each study according to nutritional, flavor-related, physicochemical, or sensory-relevant quality attributes. Overall, Table 1 shows that current evidence supports the role of resveratrol in modulating muscle protein integrity, amino acid deposition, fatty acid composition, pH, water-holding capacity, and texture-related traits. However, direct sensory-panel evidence remains scarce; therefore, the present review treats most reported flavor and texture indices as sensory-relevant proxies rather than definitive proof of improved consumer perception.

### 3.1. Effects of Resveratrol on Nutritional Quality in Fish Muscle

Nutritional components predominantly include crude protein and crude lipid, which constitute the main edible portions of fish [49]. However, to evaluate the specific nutritional value of teleost fillets for human consumption, it is crucial to look beyond crude macronutrients and examine the specific biochemical composition, such as essential amino acid profiles and the retention of health-promoting fatty acids. In juvenile southern flounder (*Paralichthys lethostigma*) fed 600 mg resveratrol/kg diet for 16 weeks, dietary resveratrol significantly reduced protein carbonylation, 4-hydroxynonenal (4-HNE), and protein ubiquitination in white epaxial muscle, indicating lower oxidative protein/lipid damage and ubiquitin-dependent protein degradation. In contrast, LC3 abundance, S6K activation, and mitochondrial volume density were not significantly affected [46]. These findings provide direct evidence of a muscle antioxidant response, although the study did not evaluate fillet composition, instrumental texture, or sensory quality. In Siberian sturgeon (*Acipenser baerii*), ten-month-old fish with an initial body weight of 248.1 ± 5.9 g were assigned to diets containing 0 or 0.16 mg resveratrol/kg for 45 days. Resveratrol did not significantly affect final body weight or percentage weight gain, but it significantly increased muscle fiber diameter. Muscle serine and histidine contents increased by approximately 11.9% and 17.0%, respectively (both *p* = 0.03), whereas total amino-acid content increased numerically by approximately 10.8% but did not reach statistical significance (*p* = 0.20). Resveratrol also significantly decreased muscle MDA and increased CAT, LDH, SOD, and total antioxidant capacity [43]. These results indicate an improvement in selected structural, nutritional, and antioxidant endpoints rather than a generalized increase in growth performance. In adult grass carp (*Ctenopharyngodon idella*), fish were fed diets containing 0, 40, 80, 160, or 320 mg resveratrol/kg for 9 weeks. Muscle crude protein increased from 18.02 ± 0.27% in the control group to 19.35 ± 1.33% at 80 mg/kg, corresponding to an increase of approximately 7.4%. Resveratrol also markedly improved oxidative stability: muscle MDA declined from 3.31 ± 0.25 to 1.53 ± 0.09 nmol/g tissue at 320 mg/kg, while protein carbonyls declined from 4.44 ± 0.43 to 3.42 ± 0.26 nmol/mg protein at 160 mg/kg. Regression analysis based on muscle protein carbonyl concentration and muscle protein content yielded estimated optimal supplementation levels of 178.33 and 198.33 mg/kg diet, respectively [45]. These different estimates demonstrate that the biologically optimal dose depends on the selected muscle-quality endpoint. Furthermore, the specific nutritional value of aquatic muscle food relies heavily on its lipid profile, particularly its abundance of health-promoting long-chain polyunsaturated fatty acids (LC-PUFAs) such as eicosapentaenoic acid (EPA) and docosahexaenoic acid (DHA). Because these essential fatty acids are highly susceptible to pre- and post-slaughter oxidation, resveratrol’s potent ability to mitigate lipid peroxidation (as observed in the flounder model) plays a vital protective role. By preserving these easily oxidizable fatty acids, resveratrol directly elevates the functional food value of the final edible fillet. Given these different effects on nutrient deposition and muscle quality in fish, it is pertinent to investigate the impact of resveratrol on the specific muscle nutritional profiles of different fish species to advance aquaculture nutrition strategies. However, the observed species-specific responses (e.g., protein increase at low doses in grass carp but myofiber diameter enhancement in sturgeon) highlight the limitation of extrapolating results across teleost species with distinct muscle metabolic profiles. Future studies should prioritize standardized comparative trials under identical rearing conditions to establish generalizable dose–response relationships for specific nutritional improvement in muscle foods.

### 3.2. Effects of Resveratrol on Flavor-Related and Sensory-Relevant Quality in Fish Muscle

Muscle flavor quality is determined by taste-active components and aroma-active components, mainly including inosine monophosphate (IMP), free amino acids, and free fatty acids [50,51]. Muscle fatty acids, particularly unsaturated fatty acids, serve as important precursors of meat aroma [51]. In rainbow trout, resveratrol has been shown to alter whole-body fatty-acid composition and affected growth/nutrient utilization [14]. Resveratrol supplementation reduced saturated fatty acid (SFA) content in grass carp muscle dose-dependently, while it elevated unsaturated fatty acids, characterized by increasing polyunsaturated fatty acid (PUFA) at 40 mg/kg, and monounsaturated fatty acid (MUFA) at 80 mg/kg [45]. However, how resveratrol influences the fatty acid composition of muscle in different fish species remains unclear and warrants investigation. Free amino acids are generally categorized into sweet, umami, and bitter amino acids according to their taste characteristics [52]. In meat ducks, resveratrol supplementation was reported to increase the content of flavor-related amino acids in the breast muscle [53]. In muscle of grass carp (*C. idella*), dietary resveratrol supplementation at 160 mg/kg significantly elevated glutamic acid (umami-related) content, while sweet-tasting amino acids (e.g., proline) and bitter-tasting amino acids (e.g., methionine, valine, isoleucine, phenylalanine, leucine, tyrosine) exhibited parabolic dose–responses peaking at 80 mg/kg [45]. Meanwhile, arginine uniquely demonstrated a concentration-dependent trend. IMP, a degradation product of ATP, is closely associated with the umami taste in fish and shellfish [54]. Although no study has yet examined the effect of resveratrol on IMP content in aquatic animal muscle, plant extracts (such as flavonoids) have been shown to increase IMP levels in chicken meat [55]. Therefore, the potential impact of resveratrol on IMP content in muscle of different fish species deserves further research. Available grass carp data indicate dose-dependent changes in selected fatty acids and amino acids; however, whether these responses are reproducible across fish species remains uncertain. Without linking these biochemical changes to actual consumer-perceived flavor in muscle food, the practical value of resveratrol for improving teleost fish palatability remains largely speculative. Despite these promising biochemical improvements, it is critical to highlight a major gap in the current literature: the striking absence of formal human organoleptic evaluations. Currently, the enhancement of fish flavor by dietary resveratrol is almost exclusively inferred through instrumental proxies, such as the quantification of umami-related free amino acids, inosinic acid, and volatile-producing fatty acids. To date, no systematic tasting panels or consumer acceptance studies have been conducted to confirm whether these micro-level biochemical shifts translate into a perceptible and statistically significant improvement in the aroma, taste, and overall palatability of the cooked fillet for human consumers. Bridging this gap through standardized sensory profiling is an urgent necessity for future research.

### 3.3. Effects of Resveratrol on the Physicochemical Quality in Fish Muscle

Fish muscle color is an important visual attribute influencing consumer acceptance, but it is not determined solely by hemoglobin and myoglobin. Depending on species, muscle type, residual blood, lipid and protein oxidation, diet-derived pigments, slaughter conditions, and postmortem handling, fillet color may vary considerably [56,57]. Currently, direct evidence regarding the effect of dietary resveratrol on fish muscle color remains scarce; therefore, any color-related effect should be regarded as a hypothesis requiring direct validation in edible fish muscle. Consequently, potential outcomes must be hypothesized based on terrestrial livestock models. For instance, in finishing pigs, resveratrol supplementation increased muscle redness at 45 min and 24 h postmortem [58], whereas in cattle, it reduced muscle lightness [59]. Whether these mammalian responses translate to the distinct myoglobin profiles of teleost muscle remains a critical hypothesis requiring empirical validation. It is critical to emphasize that because fish muscle differs fundamentally from terrestrial meats in its myoglobin concentration, structural connective tissue architecture, and protein denaturation kinetics, these terrestrial livestock and poultry responses serve only as hypothetical baselines. The distinct biochemical composition of fish muscle necessitates direct empirical validation to determine if these physicochemical improvements translate to fish species.

Texture, including hardness, is another critical quality attribute influencing consumer preference in fish [60,61]. Chewiness and hardness reflect similar textural attributes; in Furong crucian carp, resveratrol supplementation significantly increased chewiness compared with the control group [47]. Shear force is another indicator of muscle hardness. In finishing pigs, resveratrol reduced muscle shear force [58], whereas no effect was observed in broiler chickens [62]. These results indicate species-specific differences in the effect of resveratrol on muscle hardness, highlighting the need for studies across different animals. Postmortem, the loss of oxygen supply to somatic cells leads to anaerobic respiration and lactic acid production, causing a decline in pH [63]. In finishing pigs, resveratrol significantly increased pH at 24 h postmortem [58]. Dietary resveratrol supplementation with a gradient-augmented level induced a significant inverted U-shaped response in pH at 24 h postmortem, peaking at 160 mg/kg before declining in grass carp (*C. idella*) muscle [45]. These findings indicate that resveratrol can influence postmortem muscle pH in animals. Water-holding capacity (WHC) in fish muscle is inversely related to cooking loss. In meat ducks, resveratrol improved both cooking loss and drip loss, suggesting it can enhance WHC in postmortem muscle [64]. Dietary resveratrol reduced muscle cooking loss from 22.84 ± 1.58% in the control group to a minimum of 17.12 ± 1.88% at 160 mg/kg, corresponding to an approximately 25.0% reduction. Muscle shear force increased from 15.27 ± 1.16 N in the control group to 17.50 ± 1.35 N at 320 mg/kg, an increase of approximately 14.6%. The 24 h postmortem pH ranged from 6.14 to 6.24 across treatments and reached its highest numerical value (6.24 ± 0.06) at 160 mg/kg [45]. Similarly, in a 56-day feeding trial, Furong crucian carp with an initial body weight of 24.98 ± 0.05 g were assigned to diets containing 0, 150, or 300 mg resveratrol/kg. Neither supplementation level significantly affected final body weight, weight gain, specific growth rate, feed conversion ratio, survival, condition factor, or viscerosomatic index. Nevertheless, the hepatosomatic index decreased by 11.36% and 16.12% in the 150 and 300 mg/kg groups, respectively. Whole-body crude lipid decreased by 19.40% and 25.52%, and hepatic crude lipid decreased by 48.17% at 300 mg/kg. At the muscle level, 150 mg/kg resveratrol increased cohesiveness by 9.52% and chewiness by 39.22%, whereas 300 mg/kg increased cohesiveness by 9.52% and decreased adhesiveness by 51.61%. Muscle Ca^2+^-ATPase activity increased by 338.46% and 369.23%, and total antioxidant capacity increased by 65.00% and 62.50% in the 150 and 300 mg/kg groups, respectively. In addition, muscle sulfhydryl content increased by 50.00% at 150 mg/kg [47]. These results demonstrate substantial changes in selected instrumental and biochemical endpoints, although direct sensory evaluation was not performed.

Fish muscle physicochemical quality should be interpreted as a group of postmortem and storage-related indicators rather than only as color or texture. Because the present review mainly concerns pre-harvest dietary resveratrol supplementation, the strongest evidence relates to endpoints measured at harvest or shortly postmortem, whereas long-term storage and fishery-product preservation data should be considered complementary evidence. Muscle pH is a central postmortem quality parameter because it influences protein denaturation, water retention, texture, microbial stability, and consumer-perceived freshness. In grass carp, dietary resveratrol induced a dose-dependent response in 24 h postmortem pH and reduced cooking loss, suggesting a possible improvement in water-holding capacity at appropriate inclusion levels [45]. Similarly, dietary resveratrol reduced drip loss and increased chewiness in Furong crucian carp [47]. However, direct evidence remains limited, and whether resveratrol consistently regulates postmortem glycolysis, lactic acid accumulation, or pH decline across different fish species has not yet been established.

Lipid hydrolysis and oxidation are also key determinants of fish muscle and fishery-product quality [65,66]. Free fatty acids (FFAs) reflect lipase- and phospholipase-mediated lipid hydrolysis and may accelerate oxidative rancidity because free fatty acids are more susceptible to oxidation than intact lipids [67]. Peroxide value (PV) indicates the formation of primary lipid hydroperoxides, whereas thiobarbituric acid reactive substances (TBARS) mainly reflect secondary lipid oxidation products such as malondialdehyde [66,68]. Current dietary-resveratrol studies in fish rarely quantify PV or FFA in edible muscle, and most available evidence is restricted to oxidative markers such as MDA, protein carbonyls, 4-HNE, and antioxidant enzyme activities [45]. Therefore, future feeding trials should include PV, FFA, and TBARS measurements during refrigerated or frozen storage to determine whether in vivo antioxidant programming persists after harvest. Evidence from fish lipid matrices and post-harvest fishery-product models suggests that resveratrol can retard lipid oxidation [69,70], but such evidence should not be directly interpreted as proof of dietary effects in live fish.

Total volatile basic nitrogen (TVB-N) is a widely used indicator of nitrogenous spoilage caused by microbial and enzymatic degradation of proteins and non-protein nitrogen compounds. At present, direct evidence linking pre-harvest dietary resveratrol supplementation to TVB-N changes in fish fillets remains limited. However, post-harvest studies suggest that resveratrol may delay TVB-N accumulation and microbial spoilage in fishery products. For example, in a fish salami storage model, TVB-N exceeded the acceptable limit of 35 mg/100 g at the third month in the antioxidant-free control group, whereas this threshold was reached at the fifth month in the resveratrol-treated group [69]. The resveratrol group also showed lower microbial counts during storage, supporting its potential antioxidant and antimicrobial preservative role in fishery products. Similarly, a recent sturgeon-fillet model indicated that resveratrol delayed the accumulation of TVB-N, biogenic amines, and TBARS [70]. Nevertheless, these post-harvest preservation effects should be clearly distinguished from dietary supplementation studies, because the latter may regulate muscle quality through pre-harvest physiological and metabolic mechanisms.

**Figure 3:**
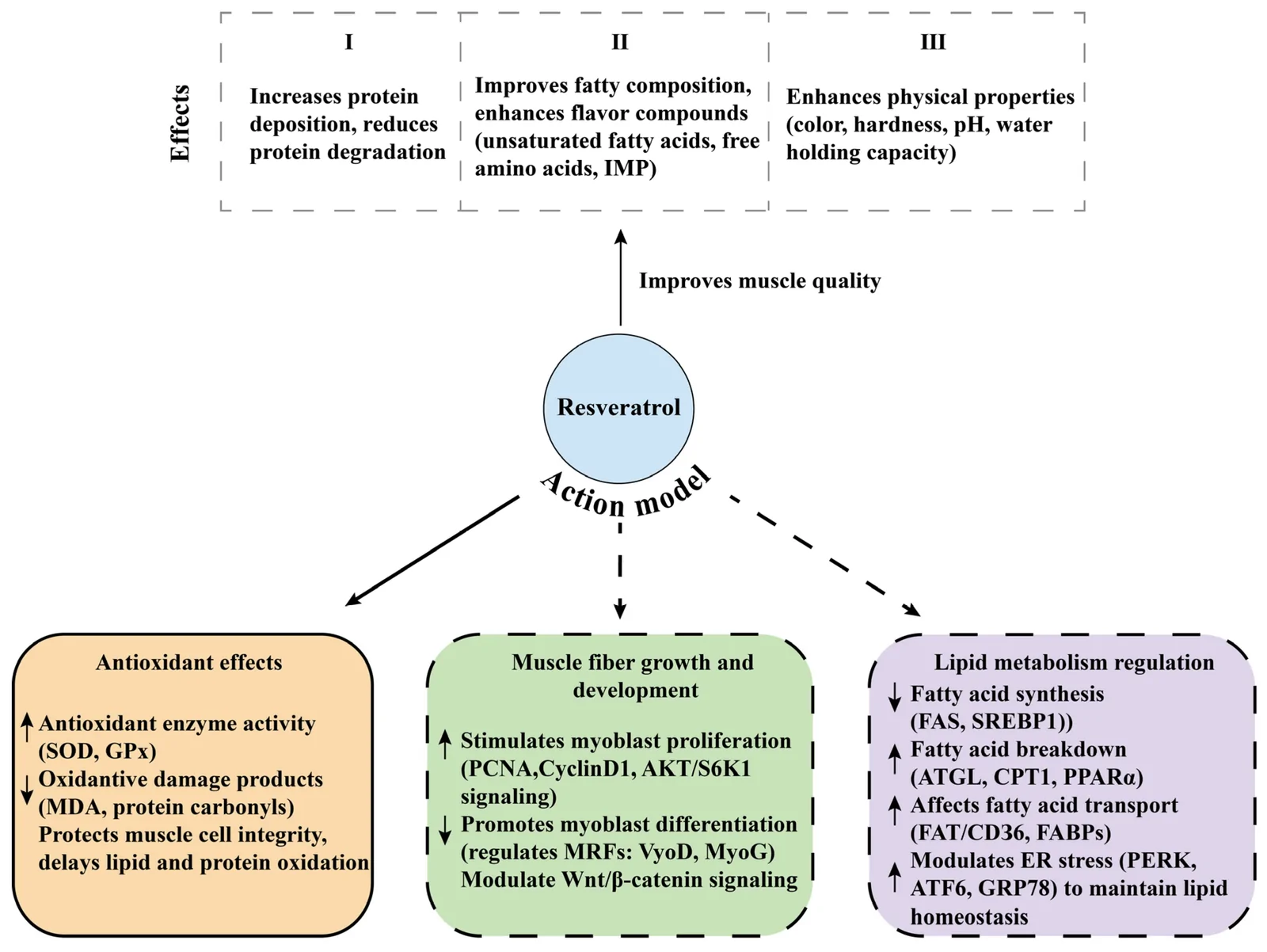
Conceptual summary of the putative relationships between dietary resveratrol and edible fish muscle quality. The upper boxes summarize nutritional, flavor-related, and physicochemical quality domains considered in this review and should not be interpreted as uniformly demonstrated effects of resveratrol. In particular, direct dietary evidence for changes in inosine monophosphate (IMP) and muscle color in edible fish muscle is currently lacking. Solid arrows indicate relationships supported by direct fish skeletal-muscle evidence. Dashed arrows indicate relationships that remain incompletely established, including mechanisms extrapolated from non-muscle fish tissues, mammalian or in vitro models, as well as direct evidence currently restricted to a single fish species or study. All dashed relationships therefore require independent validation before they can be considered broadly established mechanisms in fish muscle.

Overall, the current evidence suggests that resveratrol may influence selected physicochemical traits of fish muscle, especially pH, water-holding capacity, cooking loss, drip loss, and texture-related parameters (Figure 3). However, evidence for PV, FFA, TBARS during storage, and TVB-N remains insufficient in dietary fish studies. Future research should integrate standardized feeding trials with postmortem storage experiments, microbial counts, PV, FFA, TBARS, TVB-N, volatile-compound profiling, instrumental texture analysis, and sensory evaluation to determine whether resveratrol-induced biochemical changes translate into improved shelf-life and market quality. Figure 3 should be interpreted as an evidence-stratified conceptual summary rather than as a fully validated causal pathway. The antioxidant module is supported by direct skeletal-muscle evidence from southern flounder and Siberian sturgeon, whereas direct evidence for muscle-fiber remodeling and Wnt/β-catenin-related regulation is currently concentrated in adult grass carp. Evidence for lipid synthesis, fatty-acid catabolism, fatty-acid transport, and endoplasmic-reticulum-stress regulation is derived partly from fish liver, mammalian muscle, or in vitro models. Accordingly, the dashed arrows in these modules indicate limited cross-species validation or indirect mechanistic support rather than an absence of all experimental evidence.

## 4. Putative Mechanistic Links Between Resveratrol and Nutritional, Flavor-Related, and Physicochemical Quality of Edible Fish Muscle

The mechanisms discussed in this section should be interpreted according to the level of supporting evidence. At present, only a limited number of studies have directly examined the effects of dietary resveratrol on edible fish skeletal muscle or fillet-quality endpoints. Therefore, this section distinguishes direct fish muscle evidence from evidence derived from other fish tissues, mammalian models, in vitro systems, or studies using other dietary compounds. Mechanistic pathways such as antioxidant defense, muscle fiber remodeling, lipid metabolism, and endoplasmic-reticulum stress are discussed as putative links that may explain observed changes in muscle quality, rather than as fully validated mechanisms in fish muscle.

### 4.1. Potential Involvement of Antioxidant Defense in Resveratrol-Related Muscle Quality Changes

Oxidative stress disrupts the balance between oxidation and antioxidation in the body, resulting in decreased free radical scavenging capacity and increased free radical production. Under such conditions, proteins, lipids, and DNA in muscle tissue are damaged, compromising cellular integrity [71]. Excessive accumulation of free radicals damages membrane structures and leads to cytoplasmic loss, thereby affecting meat quality [72,73]. Malondialdehyde (MDA), the end product of lipid peroxidation, directly reflects the extent of lipid oxidative damage in muscle [74,75]. Protein carbonyls (PCs), markers of protein oxidation, are formed when free radicals react with side chains of amino acid residues such as lysine, arginine, proline, threonine, and glutamic acid in proteins [76,77]. In juvenile southern flounder (*Paralichthys lethostigma*), fed 600 mg resveratrol/kg diet for 16 weeks, skeletal-muscle protein carbonylation, 4-hydroxynonenal (4-HNE), and ubiquitination were significantly reduced, supporting lower oxidative damage and ubiquitin-dependent protein degradation. However, LC3 abundance, S6K activation, and mitochondrial volume density were not significantly affected [46]. Therefore, the study supports an antioxidant and anti-proteolytic effect in fish skeletal muscle but does not demonstrate significant regulation of LC3-associated autophagy. However, this evidence is species-specific and should not be generalized to other fish species without direct validation.

Antioxidant capacity positively influences meat quality by preventing degradation of fatty acids and amino acids, thus maintaining texture and flavor (Figure 3). Throughout evolution, organisms have developed antioxidant systems comprising enzymatic and non-enzymatic components [78]. Superoxide dismutase (SOD) rapidly removes superoxide anions, producing the less toxic hydrogen peroxide (H_2_O_2_) and oxygen [79], while glutathione peroxidase (GPx) detoxifies H_2_O_2_ [80]. Glutathione (GSH) serves as a crucial non-enzymatic antioxidant [80,81]. In turbot (*Scophthalmus maximus*) fed a soybean meal-based diet, dietary resveratrol increased hepatic SOD activity and restored the mRNA expression of antioxidant-related genes, including *sod*, *gsh-px*, and *prx6* [48]. Nevertheless, the effects of resveratrol on muscle antioxidant capacity across different fish species require further in-depth investigation. Importantly, most existing data focus on hepatic rather than muscle antioxidant responses, potentially underestimating resveratrol’s direct protective role in postmortem muscle food. Moreover, whether these hepatic antioxidant responses translate into reduced lipid and protein oxidation in edible skeletal muscle during postmortem storage or processing remains unverified.

### 4.2. Possible Role of Muscle Fiber Growth and Remodeling in Resveratrol-Related Texture Changes

Muscle fibers are the fundamental structural units of skeletal muscle. In most teleost fish, white muscle constitutes the majority of muscle mass, and its growth and development significantly influence fish flesh texture and flavor [82]. Fish muscle growth generally involves two stages: muscle fiber hyperplasia and hypertrophy. Muscle fiber characteristics include density, diameter, and cross-sectional area [60,82]. Universal fiber-diameter thresholds should not be applied across species because fiber size interacts with fiber type, oxidative phenotype, developmental stage, and sampling method [83]. Previous studies have shown that dietary resveratrol supplementation can improve muscle quality in fish. For example, in Furong crucian carp (24.98 ± 0.05 g), supplementation with 150–300 mg/kg resveratrol enhanced muscle cohesiveness, chewiness, Ca^2+^-ATPase activity, and total antioxidant capacity, while reducing lipid accumulation in the liver and whole body [47]. These findings suggest that resveratrol may positively regulate muscle quality and metabolic status. However, whether such improvements are associated with alterations in muscle fiber diameter distribution and muscle growth patterns in fish remains largely unclear and warrants further investigation.

Muscle quality is also influenced by muscle growth, with myoblast proliferation being a critical factor [84,85,86]. Cyclin D1 plays a pivotal role in promoting the G1 to S phase transition during cell cycle progression, closely associated with myoblast proliferation [87,88]. Proliferating cell nuclear antigen (PCNA) acts as a marker of DNA replication by targeting DNA polymerase during cell proliferation [89]. AKT/PKB is a broad signaling node involved in cell growth and survival [90], although that reference is not myoblast-specific. Direct fish-muscle evidence is available from adult grass carp. In a 9-week feeding trial, fish received diets containing 0, 40, 80, 160, or 320 mg resveratrol/kg. Appropriate supplementation increased the frequency of muscle fibers smaller than 20 μm, indicating enhanced myofiber hyperplasia, and the study linked this response to the stimulation of adult myoblast proliferation and differentiation and to Wnt/β-catenin-related signaling [45]. Based on quadratic regression of the frequency of muscle fibers smaller than 20 μm, the estimated optimal resveratrol supplementation level was 226.67 mg/kg diet. The same study also reported inhibition of endoplasmic-reticulum stress, which may have contributed to muscle fiber development. Thus, resveratrol-responsive myogenic and Wnt/β-catenin-related mechanisms are not entirely absent in fish. Nevertheless, the evidence currently derives principally from a single study in adult grass carp. Its reproducibility across fish species, developmental stages, feeding periods, and muscle types remains to be independently validated. In a rat model, resveratrol has been found to modulate the Akt/GSK3β signaling pathway and induce GSK3β phosphorylation [91]. Therefore, the potential effects of resveratrol on myoblast proliferation and differentiation in fish warrants further research. Although direct evidence is available in adult grass carp, long-term and cross-species validation remains necessary. Future studies should combine muscle histology, cell-proliferation markers, myogenic regulatory factors, pathway-specific interventions, transcriptomics, proteomics, and instrumental texture analysis to determine whether these molecular responses consistently translate into improved edible muscle quality.

### 4.3. Putative Regulation of Lipid Metabolism and Fatty-Acid Composition by Resveratrol

Fish tissue polyunsaturated-fatty-acid profiles strongly reflect dietary lipid sources [92]. Many fish can elongate and desaturate dietary C18 PUFA to long-chain PUFA to species-dependent extents, involving Δ5/Δ6 desaturases and elongases [93,94]. Unsaturated fatty acids play a crucial role in fish muscle flavor, serving as important precursors of aroma compounds [95]. Fatty acid content is influenced by lipid metabolism, which encompasses lipid synthesis, degradation, and transport [96,97]. Fatty acid synthesis is a key factor affecting fatty acid composition. For example, the de novo synthesis of saturated fatty acids, particularly palmitate, is primarily catalyzed by fatty acid synthase (FAS). The subsequent formation of monounsaturated fatty acids (MUFAs) from these saturated precursors is then mediated by stearoyl-CoA desaturase (SCD, also known as Δ9-desaturase) [97]. Meanwhile, polyunsaturated fatty acid (PUFA) synthesis is controlled by Δ6 desaturase (Δ6 Fad) and elongases like Elovl5. At the transcriptional level, master regulators such as sterol regulatory element-binding protein-1 (SREBP-1) orchestrate the expression of genes involved in both the synthesis and desaturation processes [93,94]. Importantly, most mechanistic data on lipid metabolism in teleosts are derived from hepatic tissues rather than skeletal muscle. For example, in high-fat diet-fed common carp (*Cyprinus carpio*), resveratrol significantly downregulated hepatic FAS and SREBP-1 expression [12]. While liver metabolism fundamentally regulates circulating lipids, directly extrapolating these hepatic responses to intramuscular fat deposition in fish muscle is highly speculative and requires targeted validation.

Beyond synthesis, lipid degradation also affects fatty acid composition, including fatty acid hydrolysis and β-oxidation [98]. Key enzymes involved in lipid breakdown include carnitine palmitoyltransferase 1 (CPT1), adipose triglyceride lipase (ATGL), and hormone-sensitive lipase (HSL) [99,100]. CPT1, as the rate-limiting enzyme of fatty acid β-oxidation, transports fatty acids into mitochondria for oxidation [99,101]. ATGL and HSL are rate-limiting enzymes in triglyceride hydrolysis and thus modulate fat deposition [102]. Peroxisome proliferator-activated receptor alpha (PPARα), highly expressed in tissues with active fatty acid metabolism, regulates genes related to fatty acid β-oxidation [103]. It has been demonstrated that resveratrol supplementation upregulated hepatic ATGL and CPT1 gene expression in high-fat diet-fed blunt snout bream (*Megalobrama amblycephala*) [44], indicating resveratrol’s potential role in promoting lipid catabolism in fish muscle, which deserves further study in muscle tissue.

Fatty acids absorbed by fish circulate in the bloodstream and are transported into muscle tissues by specific proteins such as fatty acid translocase (FAT/CD36) and fatty acid-binding proteins (FABPs) [104]. It has been found that resveratrol influenced the expression of FAT/CD36 in rat tibialis anterior muscle [105], suggesting that resveratrol may affect fatty acid transport in fish muscle as well, which remains to be elucidated.

The endoplasmic reticulum (ER) is a multifunctional organelle critical for lipid synthesis and homeostasis [106]. The activation processes of SREBP and activating transcription factor 6 (ATF6) link lipogenesis regulation with endoplasmic reticulum stress (ERS). Glucose-regulated protein 78 (GRP78) serves as a sensor of ERS and regulates the unfolded protein response proteins IRE1α, PERK, and ATF6 [107]. Silencing of GRP78 in breast cancer cells increased cellular PUFA levels, indicating its role in fatty acid composition regulation [108]. In C57BL/6 mice, scutellarin reduced hepatic lipid accumulation while suppressing, rather than activating, the IRE1α/XBP1 pathway [109]. Additionally, targeted deletion of PERK in mouse mammary epithelial cells decreased lipid synthesis and accumulation [110]. ERS can be induced by hypoxia, reactive oxygen species (ROS), and low pH, while resveratrol is known to mitigate ROS accumulation [111,112]. In a study with C57BL/6J mice fed a high-fat diet, resveratrol reduced phosphorylation of PERK and protein levels of ATF4, thereby alleviating ERS in the liver [113]. These findings suggest that resveratrol may regulate lipid metabolism through modulation of ERS, a hypothesis that requires further investigation in fish models. Collectively, while resveratrol demonstrates promising regulation of lipid synthesis, catabolism, and transport, the majority of mechanistic insights derive from liver tissue or high-fat diet models rather than skeletal muscle under standard aquafeed conditions. This tissue-specific bias, combined with limited integration of endoplasmic reticulum stress data in fish, underscores the need for muscle-targeted omics studies to fully elucidate how resveratrol optimizes fatty acid composition for both health benefits and sensory quality in teleost muscle food.

**Figure 4:**
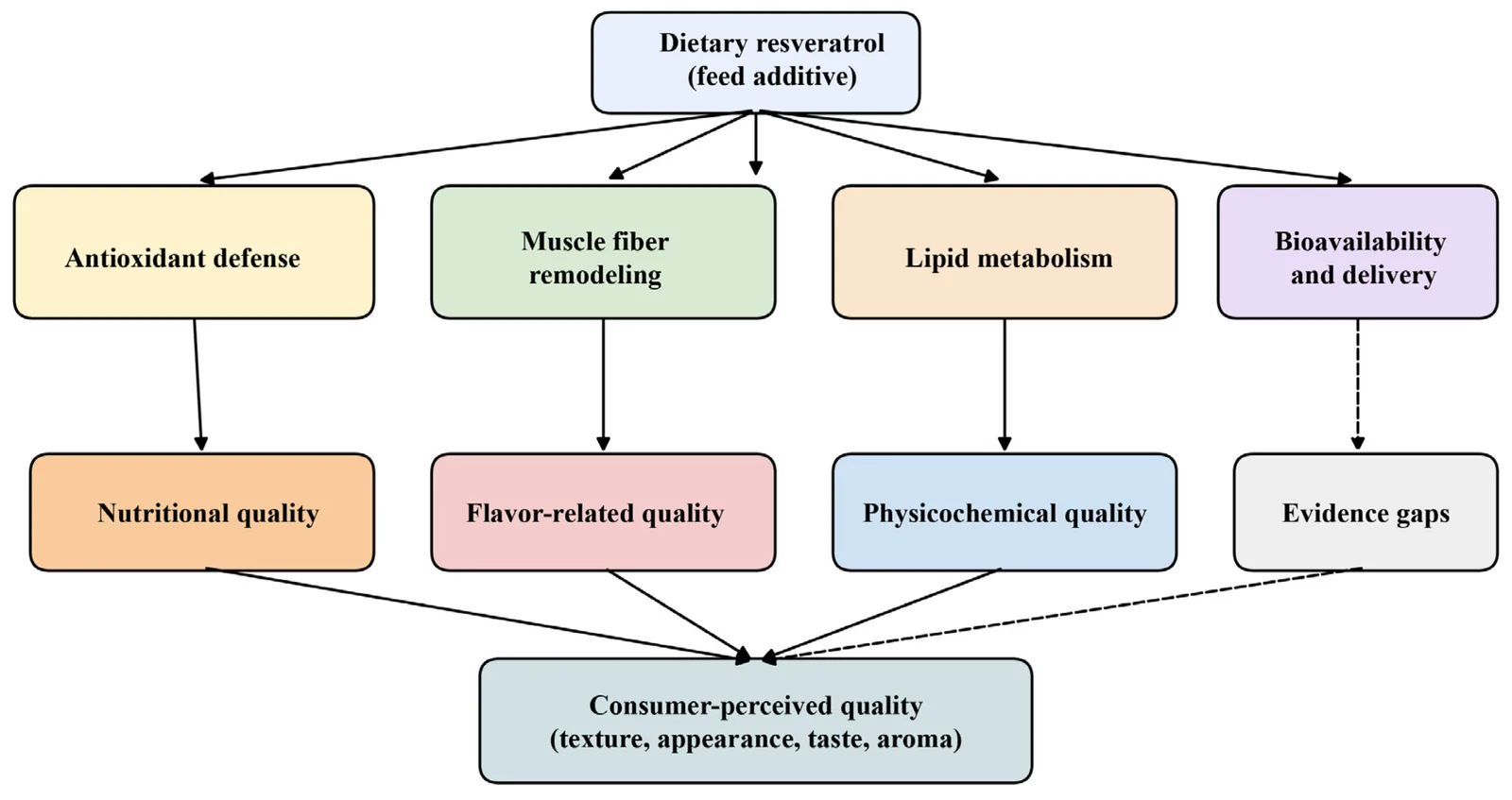
Conceptual framework linking dietary resveratrol supplementation with nutritional, flavor-related, physicochemical, and sensory-relevant attributes of edible fish muscle. The framework separates directly measured biochemical or instrumental endpoints from consumer-perceived sensory outcomes. Arrows toward sensory quality indicate hypotheses that require validation by volatile compound profiling, trained-panel sensory profiling, and consumer acceptance testing.

Based on the evidence summarized above, a conceptual framework was constructed to clarify how dietary resveratrol may regulate fish muscle-food quality through interconnected physiological pathways (Figure 4). This framework separates upstream mechanisms from measurable quality attributes and from final consumer-perceived sensory outcomes, thereby avoiding overinterpretation of biochemical proxy data as direct sensory evidence.

Table 2 further summarizes the mechanistic links between resveratrol-responsive pathways and muscle-quality outcomes. This synthesis highlights that antioxidant protection, muscle fiber remodeling, lipid metabolic regulation, and improved delivery strategies may jointly contribute to nutritional and sensory-relevant quality, but that several links remain inferential and require validation using muscle-targeted omics, volatile-compound profiling, instrumental texture analysis, and trained sensory panels.

## 5. Challenge and Future Prospects

As comprehensively detailed in Section 2.2, despite exhibiting a high oral absorption rate, resveratrol is subject to rapid and extensive phase II metabolism, plasma protein binding, and enterohepatic recirculation, which collectively result in exceptionally low in vivo bioavailability. This severe limitation in bioavailability remains the primary bottleneck for its practical application. Overcoming this challenge requires innovative formulation strategies to enhance its utilization. Promising approaches include: (1) synergistic use with other phytochemicals: co-administration with other plant-derived compounds can protect resveratrol from rapid metabolism and thereby increase its bioavailability [114,115]; (2) exploitation of resveratrol metabolites: Resveratrol sulfates can deliver resveratrol more effectively to target tissues in vivo, and their conjugated forms are more stable. Resveratrol sulfate has been shown to regenerate resveratrol in mice, maintaining physiological concentrations in plasma and tissues [116]; (3) use of natural resveratrol analogs, such as resveratrol trimethyl ether [117] and oxyresveratrol [118], which exhibit faster absorption and slower clearance than resveratrol; (4) development of novel formulations such as nanoparticles, liposomes, capsules, and polymeric micelles. In addition, polymeric micelles (~52 nm) prepared via the thin-film dispersion method can enhance encapsulation efficiency, drug-loading capacity, and stability, while providing sustained-release properties that improve the in vivo stability and bioavailability of resveratrol [119,120,121].

From the perspective of muscle food production, overcoming resveratrol’s bioavailability challenges through innovative delivery systems is crucial for its practical application as a feed additive to consistently improve the nutritional profile, flavor, and texture of fish muscle destined for human consumption. Critically, the current literature lacks head-to-head comparisons of resveratrol with other polyphenols (e.g., quercetin, curcumin) commonly used in aquafeeds, making it difficult to assess its relative efficacy and cost-effectiveness as a muscle food quality enhancer. Furthermore, the optimal dosage of resveratrol in aquatic animals has not yet been standardized. The synergistic or antagonistic interactions between resveratrol and other established aquafeed antioxidants (such as vitamin E, astaxanthin, curcumin, and quercetin) require systematic comparative evaluations to establish its relative efficacy and cost-effectiveness. Furthermore, from a food science perspective, future investigations must bridge the gap between in vivo nutritional interventions and post-harvest realities. This includes evaluating feed-processing stability under commercial extrusion conditions, conducting rigorous edible-tissue safety and regulatory assessments, and performing extended shelf-life trials to monitor volatile compound evolution, instrumental texture dynamics, and sustained lipid/protein oxidation during prolonged frozen storage. Finally, the transition of resveratrol from an experimental laboratory supplement to a commercially viable aquafeed additive requires rigorous real-world production validation. The prospects for implementing this approach at an industrial scale depend heavily on resolving the economic feasibility and feed-processing stability of resveratrol. Commercial extrusion processes involve high heat and pressure, which may degrade unprotected resveratrol before ingestion. Furthermore, while the specific nutritional value of the raw material (fresh fillet) is improved, the extent to which these benefits, such as enhanced antioxidant capacity and LC-PUFA retention, persist through industrial processing (e.g., freezing, smoking, or canning) remains completely unexplored. Therefore, future translational research must incorporate pilot-scale farm trials, cost–benefit economic analyses, and complete farm-to-fork evaluations to definitively establish resveratrol’s utility in modern aquaculture production.

## 6. Conclusions

Resveratrol is a promising dietary polyphenol with potential relevance to edible fish muscle quality, but current evidence remains limited and uneven. Available studies suggest possible effects on nutritional composition, oxidative stability, pH, water-holding capacity, texture-related traits, and fatty-acid or amino-acid profiles in selected fish species. However, many proposed mechanisms are still based on indirect evidence from other fish tissues, mammalian models, in vitro systems, or general antioxidant biology. Direct evidence linking resveratrol supplementation with improved fish fillet quality, storage stability, and consumer-perceived sensory attributes is still insufficient. Future research should prioritize standardized dose–response trials, direct muscle-quality measurements, PV, FFA, TBARS, TVB-N, volatile compound profiling, trained-panel sensory evaluation, and delivery-system optimization. These studies are necessary before resveratrol can be reliably recommended as a practical aquafeed additive for improving fish muscle food quality.

## Figures and Tables

**Table 1:** Experimental evidence relevant to dietary resveratrol and edible fish muscle quality: direct muscle endpoints and indirect mechanistic evidence.

Species/ Model	Resveratrol Treatment	Muscle-Food Endpoint	Major Reported Change	Quality Category	Evidence Limitation	Reference
Southern flounder	Dietary supplementation	Protein carbonyls, 4-HNE, ubiquitination	Lower protein/lipid oxidation and ubiquitination	Nutritional stability; oxidative quality	No trained sensory panel; biochemical endpoints only	[46]
Siberian sturgeon	Dietary supplementation	Myofiber diameter, amino acids, MDA, CAT, LDH, SOD, T-AOC	Larger myofibers; higher Ser and His; lower MDA; higher antioxidant capacity	Nutrition; texture-related structure	Species-specific response; sensory validation lacking	[43]
Grass carp	Dietary supplementation	Protein/lipid, FA, amino acids, cooking loss, shear force, pH	Higher protein and selected UFA/amino acids; lower cooking loss; higher shear force	Nutritional, flavor-related, physicochemical	Dose–response is nonlinear; consumer perception not measured	[45]
Rainbow trout	Dietary supplementation	Growth, nutrient utilization, fatty-acid composition	Modulated fatty-acid profile and nutrient utilization	Flavor-related lipid precursors; nutrition	Muscle sensory endpoints not directly tested	[14]
Furong crucian carp	Dietary supplementation	Cohesiveness, chewiness, Ca^2+^-ATPase, T-AOC	Higher cohesiveness, chewiness, Ca^2+^-ATPase and T-AOC	Physicochemical texture; metabolic quality	Instrumental texture rather than human sensory evidence	[47]
Turbot	Dietary supplementation	Hepatic SOD activity; *sod*, *gsh-px*, *prx6* expression	Higher SOD activity and restored antioxidant-gene expression	Mechanistic support for antioxidant-related muscle-quality hypotheses	Hepatic antioxidant evidence; direct skeletal-muscle validation is still needed	[48]
Common carp	Dietary supplementation	Hepatic FAS, SREBP-1	Lower hepatic FAS and SREBP-1	Mechanistic support for lipid-metabolism-related muscle-quality hypotheses	Hepatic/high-fat-diet evidence cannot be directly equated with intramuscular lipid remodeling	[12]
Blunt snout bream	Dietary supplementation	Hepatic ATGL, CPT1	Higher hepatic ATGL and CPT1	Mechanistic support for lipid-catabolism-related muscle-quality hypotheses	Hepatic evidence requires validation in skeletal muscle and fillet-quality endpoints	[44]

**Table 2:** Conceptual synthesis of mechanism-quality links and remaining validation needs.

Mechanistic Module	Representative Markers/Pathways	Most Relevant Quality Outcomes	Current Evidence Base	Key Validation Needed	Reference
Antioxidant defense	SOD; *sod*, *gsh-px*, *prx6*; MDA, PC, 4-HNE	Oxidative stability; protection against lipid/protein oxidation	Direct muscle oxidative-damage evidence in southern flounder; hepatic antioxidant evidence in turbot	Link muscle antioxidant responses with postmortem lipid/protein oxidation and sensory rancidity	[46,48]
Muscle fiber growth/remodeling	PCNA, Cyclin D1, AKT/S6K1, MyoD, Myf5, MyoG, Wnt/β-catenin	Myofiber structure; texture; muscle protein accretion	Structural evidence in Siberian sturgeon and grass carp; texture evidence in Furong crucian carp; mechanistic evidence mainly from grass carp	Long-term, multi-species histological and texture validation	[43,45,47]
Lipid synthesis and catabolism	FAS, SREBP-1, CPT1, ATGL, HSL, PPARα	Intramuscular lipid deposition; SFA/MUFA/PUFA balance; flavor-volatile precursors	Mostly hepatic or high-fat-diet evidence; some muscle fatty-acid outcomes	Muscle-targeted lipidomics under standardized aquafeed conditions	[12,14,44,45]
Fatty-acid transport and ER stress	FAT/CD36, FABPs, GRP78, PERK, ATF6, IRE1α	Fatty-acid partitioning, oxidative stability, and possible flavor precursor availability	Largely extrapolated from mammalian or non-muscle evidence	Teleost skeletal-muscle validation and causal intervention studies	[105,106,107,108,109,110,111,112,113]
Sensory translation	Free amino acids, fatty acids, pH, WHC, shear force	Appearance, aroma, taste, texture, overall acceptability	Mostly biochemical/instrumental proxies in fish	GC-MS volatile profiling plus trained sensory panel and consumer acceptance testing	[45,47]

## Data Availability

No new data were created or analyzed in this study.
